# Experiences of Orthopaedic Camp in a Mobile Surgical Unit (Life Line Express) in Central Part of India

**DOI:** 10.5704/MOJ.1311.009

**Published:** 2013-11

**Authors:** Gajanan Deshmukh, HKT Raza

**Affiliations:** Department of Orthopaedics, Netaji Subhash Chandra Medical College, Jabalpur, India; Department of Orthopaedics, Netaji Subhash Chandra Medical College, Jabalpur, India

## Abstract

We present our experience organizing an orthopaedic camp in rural part of India in a mobile surgical unit (Life Line Express) on a train. The camp was held for 15 days from 25th may to 10th June 2009. We performed deformity correction surgeries; corrective plaster castings and follow up the cases for the next six month. We assess the pros and cons of this orthopaedic camp on a train where minor and major procedures were carried out.

## Introduction

French surgeon Nicholas Andre in 1741 coined the term “orthopaedics”, meaning straight child. Thus, orthopaedics has roots firmly embedded in the art and science of preventing and correcting deformity. In India majority of the population live in rural areas, so most of the disabled and crippled persons are unable to reach out to the tertiary health centres for corrective surgeries and other aids. Without corrective surgery, these children are condemned to a lifetime of isolation and suffering. Taunted and tormented for their disfigurement, they cannot attend school, hold a regular job or get married. Many are even abandoned or killed at birth.

On 16th July 1991 the dream came true for neglected people when Impact India established the 1st hospital on wheels in the world in the form of LifeLine Express. This project had been developed in collaboration with the Indian Railway and Health Ministry. Lifeline Express had arrived at the railway station Jabalpur (Figure 3a) to establish the camp in various disciplines such as ENT, Ophthalmology, Dentistry, Surgery, Plastic surgery and Orthopaedics. In Lifeline Express the orthopaedically disabled were operated from 25th may to 10th June 2009.

We held an Assessment Camp in which we had run the outpatient clinic in the Regional Spinal Injury Centre as shown in fig 5. At that time patients were checked and assessed whether their deformity could be corrected. Suitable patients were admitted to the Regional Spinal Injury Centre Jabalpur (Figure 4). A list of surgically fit patients were prepared and consultants from the Department of Orthopaedics and Regional Spinal Injury Centre further examined the patients. Deformity and muscle power in the limbs were assessed and treatment options discussed. The operation theatre list was then finalised. After informed consent was taken, we performed injection of tetanus toxoid, and checked for xylocain sensitivity. Each patient was tagged with sticker indicating name, date of operation and registration number. Preoperative photographs were taken. These patients were then transported to the Lifeline Express.

**Fig. 1:**
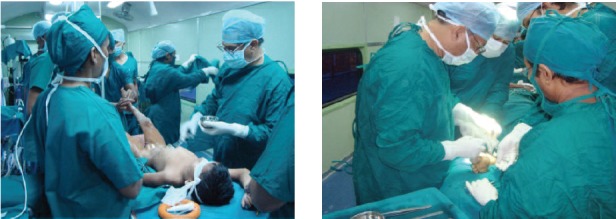
: Three operating tables running at a time.

Two hundred and eleven orthopaedically handicapped patients were operated in the Life Line Express from 25th May to 10th June 2009 by surgeons from the Department of Orthopaedics Medical College Jabalpur and Regional Spinal Injury Centre. Some orthopaedic surgeons from private sector participated in the project. At any point of time three operating lists were running as shown in fig 1. Every day we started operations at 9:00 a.m. and ended at 6:00p.m. On an average, we had operated upon 14 patients per day as shown in table I. Total number of procedures done in Life line Express from 25th May 2009 to 10th June 2009 was 402 (Table III). All patients and their accompanying persons were provided meals at the Regional Spinal Injury Centre, at a cost of rupees 20 /each person per meal plate. This food supply was continued for 2 months (Figure 7).

## Observation

To conduct the operative camp of such a large in mobile surgical unit requires proper planning. In the beginning, we organised the pre assessment clinic during which the patients were examined, registered admitted to the local preoperative wards.

As the train reached Jabalpur (which is the divisional headquarters with medical college, Regional Spinal Injury Centre, intensive care and blood banks were available), we are able to perform both minor and major surgeries. While the train was in station at Jabalpur we had full support from the local government.

Lifeline Express has the facility for running three operating lists at the same time. It Has its permanent staffers such as the cook, technician in-charge of maintenance, pathology services, an operating theatre assistant, and computer facilities.

The train has its own sterilization system in the form of autoclave and very few of our cases had post-operative infection. Operative camp of such scale requires good infrastructure. To hold such camp, one should have the back up of a tertiary health centre.

Consultants from the Regional Spinal Injury Centre assessed the patients. We had paid special attention to avoid problems of wrong identification of patients, wrong side, wrong procedures, and wrong follow-up. Patients were given identification tags before being transported to the train for surgery and checked again in the OT.

As the day progresses, the sterilization status of the operation theatre may not be maintained. This requires major surgeries being done on priority at the start of each day, so that the infection rate in these cases would be minimized.

We followed up the cases in a systematic manner over a period of six month. Some cases of major surgeries are still being followed-up up to the present time, and the results have been good.

## Discussion

The goal of every medical mission is to fulfil a child's greatest wish: “The Chance to be Normal” 2 A unique and successful medical mission model is the foundation for providing safe surgeries for children in remote areas of the world and for working towards a long-term sustainable solution. Every year 35,000 children in India are born with cleft lips and/or palate. Though completely treatable, less than half get the treatment they desperately need – only because they are too poor 2. Without corrective surgery, these children are condemned to a lifetime of isolation and suffering.

The Department of Orthopaedics Jaipur4 has mobile surgical unit which conduct surgery for physically challenged people. They are doing various surgeries such as muscle release, tendon transfer, osteotomies for post-polio deformities, and for varus ,valgus and recurvatum deformities of the knee, Z-plasty for flexion contractures of finger , excision of accessory fingers in -polydactyly, and so on.

It is frequently asked whether Mobile Surgical Units (MSU)^5^ will still be required with improved medical facilities. Such views stem from ignorance about ground realities in countryside or rural areas. In every MSU camp, a huge number of patients come to the camp irrespective of the location, even when such camps are organized at district headquarters or regions where medical facilities are said to be good. This is proof of the trust that people have in MSUs, which incidentally provides totally free service.

Dr Rahul Khare, Dr AK Agarwal, Dr Ratnesh Kumar, (2004)^6^ studied twenty two surgical polio camps organized in 8 districts of Uttar Pradesh and Madhya Pradesh from January 2000 to May 2006. Over 8000 children were screened, and divided into three groups: for physiotherapy, calipers and those who needed surgical correction for their deformities. 3370 patients were advised physiotherapy, 2920 were given calipers while 1250 patients were operated on. By and large bony operations were avoided. Ninety-six percent of cases had full correction of deformities and only 4% of cases needed further physiotherapy before fitting of orthoses. Such rehabilitative surgical polio camps offer a ray of hope for these illiterate, ignorant and unfortunate patients, and the way to an independent respectable life.

Publicity plays a major role in the success of the camp so they advise organizers and government machinery of the area to reach even remote places but the reality is that overpublicity is as dangerous as lack of publicity, as people think that every condition will be treated in these camps. Some patients requiring cardiac and tumors surgeries also come to the MSU but as these surgeries are possible only at tertiary care centers, but they are disappointed that they cannot be treated.

**Table I:**
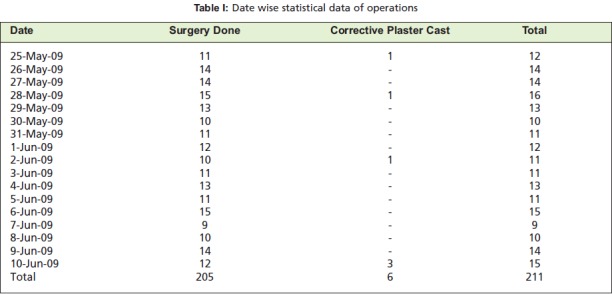
: Date wise statistical data of operations

**Table II:**
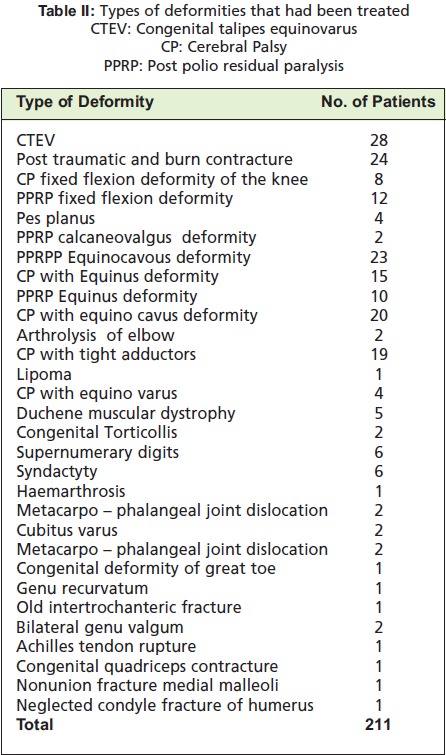
: Types of deformities that had been treated CTEV: Congenital talipes equinovarus CP: Cerebral Palsy PPRP: Post polio residual paralysis

**Table III:**
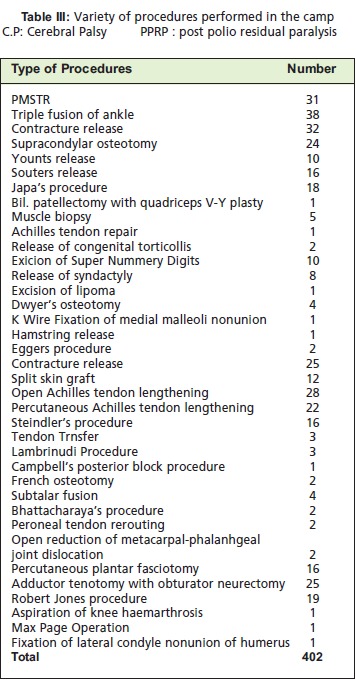
: Variety of procedures performed in the camp C.P: Cerebral Palsy PPRP : post polio residual paralysis

**Fig. 2:**
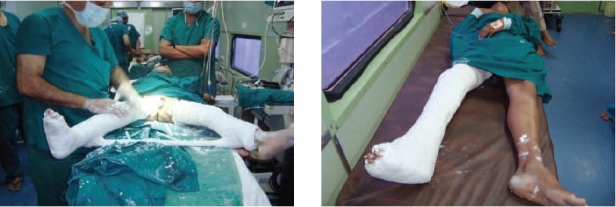
: Correction plaster cast after surgery.

**Fig. 3a:**
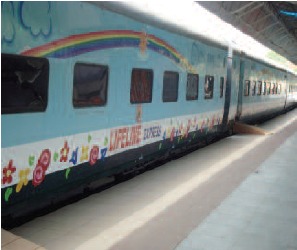
: Life line at Jabalpur railway station.

**Fig. 3b:**
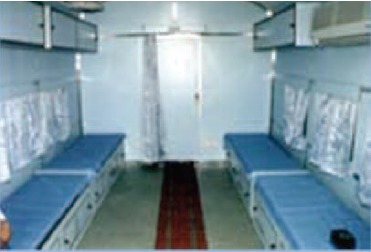
: recovery room.

**Fig. 3c:**
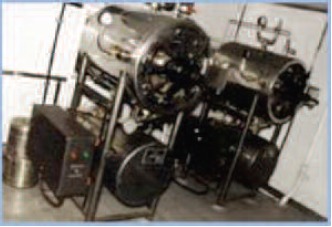
: Autoclave

**Fig. 4:**
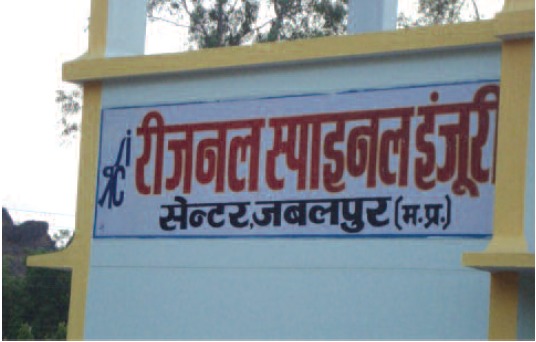
: Spinal regional injury centre near medical college Jabalpur where patients were admitted.

**Fig. 5:**
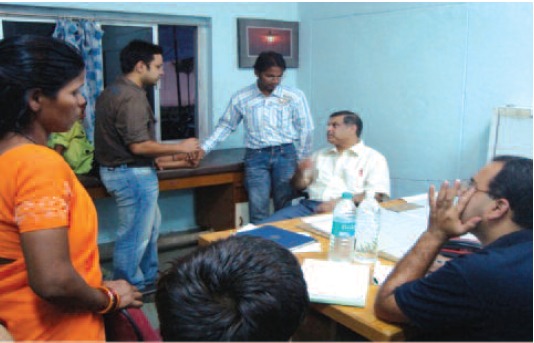
: Pre operative assessment by consultants and residents

**Fig. 6:**
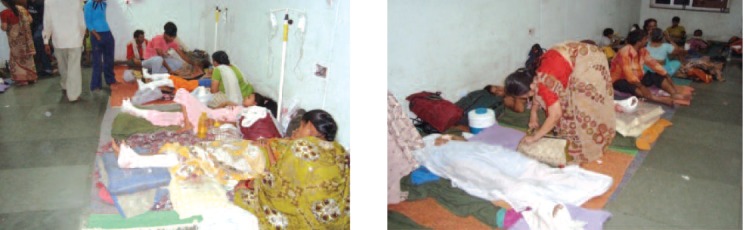
: Post operative wards.

**Fig. 7:**
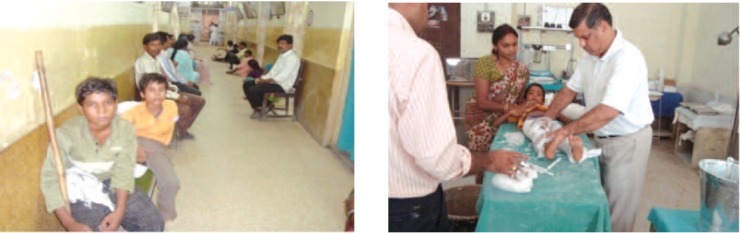
: Follow up at N.S.C.B.Medical collge Jablpur.

**Fig. 8:**
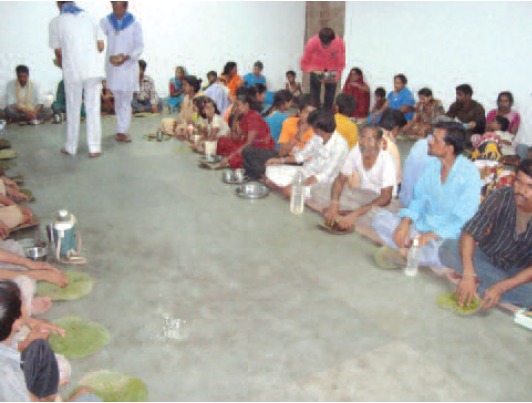
: Food supply organised Rotatory club Jabalpur.

## Conclusion

The mission of Lifeline Express is to eradicate avoidable blindness, deafness and physical handicaps. Perhaps the greatest advantage Lifeline Express has over other health services for the poor is its ability to reach "the doorstep of the patient”. The physically handicapped provide the opportunity for Orthopaedic surgeons to conduct careful physical examination, muscle charting, gait evaluation, soft tissue handling and much more.

Surgeons from nearby locations come to attend the camps and work with the mobile teams. Experienced surgeons from medical colleges also attend these camps regularly as they take it as a great opportunity to improve their professional skills.

To improve the functioning of mobile surgical unit better equipment and instruments as well as the latest techniques should be introduced. In addition, there should be a strong back up from nearby teaching institution for necessary investigations, which cannot be done in the camps. There should be proper publicity to avoid disappointing the poor community of patients seeking tertiary level care, which the camp is not equipped to provide. To conduct the successful operative camp in large magnitude, it requires proper planning and assessment, with tertiary health care backup and proper follow-up where complication like infection can be taken care of. Preferably those surgeons operating at camp should provide the follow up. Camp of this magnitude requires good infrastructure like in patients’ wards, dressing room, plaster room, operating theatre.

Poor and needy people get an opportunity to have the tertiary health care by attending follow up in tertiary centres. But this could be facilitated when MSU is conducting camp close to tertiary health centres. Orthopaedic deformity correction camps offer hope for illiterate, ignorant, unfortunate patients to lead an independent respectable life. Orthopaedic departments across the country are already overburdened with trauma patients. Physically challenged people are neglected and do not get priority for surgery. Being poor they cannot seek corrective surgery in private hospitals. MSU provides an avenue of healthcare opportunity for these people.
